# A Robust INS/USBL/DVL Integrated Navigation Algorithm Using Graph Optimization

**DOI:** 10.3390/s23020916

**Published:** 2023-01-12

**Authors:** Peijuan Li, Yiting Liu, Tingwu Yan, Shutao Yang, Rui Li

**Affiliations:** 1 Industrial Center, School of Innovation and Entrepreneurship, Nanjing Institute of Technology, Nanjing 211167, China; 2 School of Automation, Nanjing Institute of Technology, Nanjing 211167, China

**Keywords:** USBL, DVL, inertial navigation, underwater navigation, factor graph

## Abstract

The Autonomous Underwater Vehicle (AUV) is usually equipped with multiple sensors, such as an inertial navigation system (INS), ultra-short baseline system (USBL), and Doppler velocity log (DVL), to achieve autonomous navigation. Multi-source information fusion is the key to realizing high-precision underwater navigation and positioning. To solve the problem, a fusion scheme based on factor graph optimization (FGO) is proposed. Due to multiple iterations and joint optimization of historical data, FGO could usually show a better performance than the traditional Kalman filter. In addition, considering that USBL and DVL are usually heavily influenced by the environment, outliers are often present. A robust integrated navigation algorithm based on a maximum correntropy criterion and FGO scheme is proposed. The proposed algorithm solves the problem of multi-sensor fusion and non-Gaussian noise. Numerical simulations and field tests demonstrate that the proposed FGO scheme shows a better performance and robustness than the traditional Kalman filter. Compared with the traditional Kalman filtering, the positioning accuracy is improved by 5.3%, 9.1%, and 5.1% in the east, north, and height directions. It can realize a more accurate navigation and positioning of underwater multi-sensors.

## 1. Introduction

Underwater navigation has always been a challenging research hotspot [1]. Navigation based on satellite signals is often unavailable due to the limited underwater environment. An inertial navigation system (INS) is an autonomous navigation model [2], free from environmental interference. It can be applied to underwater navigation. However, inertial navigation has the problem of a cumulative divergence of errors over time. Error suppression of inertial navigation is always a difficult problem in underwater navigation and positioning.

Acoustic navigation is an effective underwater navigation scheme. The ultrashort baseline system (USBL) is an alternative navigation method. Therefore, inertial navigation assisted by an ultra-short baseline [3,4] has been a research hotspot of underwater navigation in recent years. Back in 2006, Morgado first proposed an INS/USBL integrated navigation model [5], in which a simplified INS mechanization is used to conduct the state model. In 2013, experiments verified the positioning accuracy of integrated navigation [6]. However, the mathematical model of INS is simplified, and the accuracy of integrated navigation is limited. To improve the accuracy of underwater navigation based on USBL, Zhang proposed a loosely coupled integrated navigation model based on a high-precision strap-down inertial navigation mechanical model [7]. In [8], Zhang also proposed a tightly coupled model based on relative measurement information, which has a better performance than the loosely coupled model. However, the positioning range of the USBL is limited.

In addition to USBL, another common method of underwater navigation is the Doppler velocity log (DVL). The research on INS/DVL focuses mainly on error calibration [9], initial alignment [10], and fusion algorithm [11]. For example, in [9], an iterative method is designed to calibrate the scale factor and installation angle. The error calibration is the basis of integrated navigation. In [11], the INS/DVL is assisted by ZUPT to reduce the drift. To improve the INS/DVL integrated navigation accuracy, Wang proposed a correction method by INS attitude dynamics [12]. Different from the traditional velocity-matching integrating scheme, Liu proposed an integrated model based on DVL beam measurements [13]. To deal with the interference of outliers, an M-estimation-based interacting multiple model for INS/DVL navigation is proposed [14]. Different from USBL, DVL is not restricted by distance and can realize navigation and positioning functions in any environment. However, due to the velocity-matching navigation model, its positioning accuracy is not as high as the USBL.

Considering the advantages of USBL and DVL, respectively, an INS/DVL/USBL integrated navigation system is a feasible scheme for Autonomous Underwater vehicles (AUV). Integrated navigation filtering methods can be divided into centralized filtering [15] and federal filtering [16]. In [15], a grid INS/USBL/DVL integrated navigation algorithm for polar navigation based on centralized filtering was proposed. A vector fault detection method was designed to improve the robustness of the system. Different from the centralized filtering, an INS/USBL/DVL integrated navigation model based on federal filtering is proposed in [16]. To isolate the faulty sub-filter, it improves its fault tolerance. In [17], experiments showed that the integrated navigation system had been proven to perform better than INS/USBL or INS/DVL systems. However, both of them are based on the filter scheme. The improvement in accuracy is very limited.

The optimization-based method is widely used in simultaneous localization and mapping (SLAM) systems [18]. However, the application of a factor graph is not limited to SLAM but also has many applications in other localization fields. As early as 2012, factor graph optimization (FGO) was applied to global navigation satellite system (GNSS) positioning [19]. Since then, FGO has been successfully used to improve the positioning accuracy of GNSS [20,21]. In the GNSS/INS integrated navigation system, Wen first verified that the positioning accuracy based on FGO was better than that of the Kalman filter under tightly coupled conditions [22].

In view of the advantages of FGO compared with the Kalman filter in the field of underwater navigation, an FGO-based fusion scheme is proposed for the underwater INS/DVL/USBL system. Due to multiple iterations and a large amount of historical data, a higher positioning accuracy can be obtained. In addition, considering the effect of outliers, we add a robust algorithm based on the maximum entropy criterion to the factor graph model.

The contributions of our work are summarized in three points:

(1) An optimization-based INS/USBL/DVL integrated navigation model is proposed. Measurement factors such as the IMU factor, USBL factor, and DVL factor are designed. It could play a superior performance to the traditional filter method.
(2) A robust estimation method combining the maximum entropy criterion and FGO is proposed. It can overcome the influence of outliers and improve the robustness of the integrated system.
(3) Experiments have been conducted to show that it can achieve a more accurate and robust localization than the traditional methods for underwater positioning.

## 2. System Model

The coordinate systems used in the paper include a body frame (b-frame), navigation frame (n-frame), USBL frame (u-frame), and geographic frame (g-frame). Detailed definitions can be found in [8].

**Figure 1:**
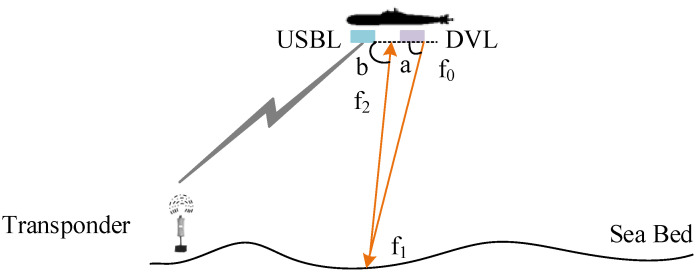
Sketch of AUV positioning.

The AUV is equipped with a USBL and DVL, as shown in Figure 1.

The transducer of USBL on AUV sends an acoustic signal to the underwater transponder, which receives the signal and returns it to the transducer. The slant distance (*r*) is calculated from the round-trip time of the acoustic signal. The bearing angles (α,β) are measured according to the angle-of-arrival.

Thus, the relative position of the transponder in the u-frame is calculated by:

(1) $$t^{u}=\left[\begin{array}{c} t_{u,x}\\ t_{u,y}\\ t_{u,z} \end{array}\right]=\left[\begin{array}{c} r\cos\alpha \\ r\cos\beta \\ -\sqrt{r^{2}-{\left(rcos\alpha \right)}^{2}-{\left(rcos\beta \right)}^{2}} \end{array}\right]$$

The transponder position ($t^{g}={\left[\begin{array}{ccc} \lambda_{t} & L_{t} & h_{t} \end{array}\right]}^{T}$) is known. Thus, the position of AUV ($p^{g}$) can be obtained:

(2) $$p^{g}=t^{g}-C_{n}^{g}C_{b}^{n}C_{u}^{b}t^{u}$$

where $C_{u}^{b}$ denotes the installation error matrix from the u-frame to b-frame. It can be calibrated in advance [23]. $C_{b}^{n}$ is the attitude matrix.$\;C_{n}^{g}$ denotes the coordinate transformation matrix from the n-frame to g-frame, which is represented as follows:

(3) $$C_{n}^{g}=\left[\begin{array}{ccc} 0 & \frac{1}{R_{M}+h} & 0\\ \frac{\sec L}{\left(R_{N}+h\right)} & 0 & 0\\ 0 & 0 & 1 \end{array}\right]$$

where the definitions of $R_{N}$ and $R_{M}$ can be seen in [2]. h denotes the height.

The DVL also emits sound waves to the ocean floor, as Figure 1 shows. Assume that the frequency of transmitting acoustic signals is $f_{0}$. The angle between the beam and the horizontal plane is a. The frequency of sound waves as they reach the seafloor is $f_{1}$. The frequency is $f_{2}$ when it reaches AUV again after reflection, and the angle between the beam and the horizontal plane is b. According to the principle of Doppler frequency shift, $f_{1}$ can be obtained:

(4) $$f_{1}=\frac{c0\;\cdot \;f_{0}}{c0-v_{y}cosa}$$

where c0 is the sound speed. $v_{y}$ is the moving speed in the *y* direction in the b-frame.

$f_{2}$ can be obtained:

(5) $$f_{2}=\frac{f_{1}\left(c0+v_{y}cosb\right)}{c0}=\frac{f_{0}\left(c0+v_{y}cosb\right)}{c0-v_{y}cosa}$$

Thus, the velocity of AUV in the b-frame can be obtained [24]:

(6) $$V^{b}=\left[\begin{array}{c} 0\\ \frac{c0\;\cdot \;f_{d}}{2f_{0}cosa}\\ 0 \end{array}\right]$$

where $f_{d}=f_{2}-f_{0}$.

## 3. FGO-Based Integrated Navigation Algorithm

In this section, we will propose a robust integrated navigation algorithm based on FGO and the maximum entropy criterion.

In an integrated navigation system, the state estimation problem can be formulated as:

(7) $$\tilde{x}=\mathrm{argmax}\underset{k,i}{\prod }P\left(z_{k}|x_{k}\right)\underset{k}{\prod }P\left(x_{k}|x_{k-1}\right)$$

where $\tilde{x}\;$ denotes the optimal state estimation. $x_{k}$ represents the system state at time *k*. $z_{k}$ represents the measurement vector at time *k*.

In FGO, we combine the measurements of all historical nodes, and the optimal estimation problem can be treated as a maximum likelihood estimation (MAP) problem. It can be obtained:

(8) $$\tilde{x}=\mathrm{argmax}\underset{k,i}{\prod }P\left(z_{k}|x_{k}\right)\underset{k}{\prod }P\left(x_{k}|x_{k-1}\right)$$

where $f_{j}\left(x_{j}\right)$ is the *j*-th factor. It can be formulated as:

(9) $$f_{j}\left(x_{j}\right)\propto \exp\left(-\|h_{j}\left(x_{j}\right)-z_{j}\|{}_{\Sigma_{j}}^{2}\right)$$

where $h_{j}\left(x_{j}\right)$ is the measurement function and $z_{j}$ is the *j*-th measurement. $\Sigma_{j}$ is the covariance matrix.

Thus, (8) is equal to the following [25]:

(10) $$\tilde{X}=\underset{x}{\mathrm{argmax}}\left(\underset{j}{\sum }\|h_{j}\left(x_{j}\right)-z_{j}\|{}_{\Sigma_{j}}^{2}\right)$$

**Figure 2:**
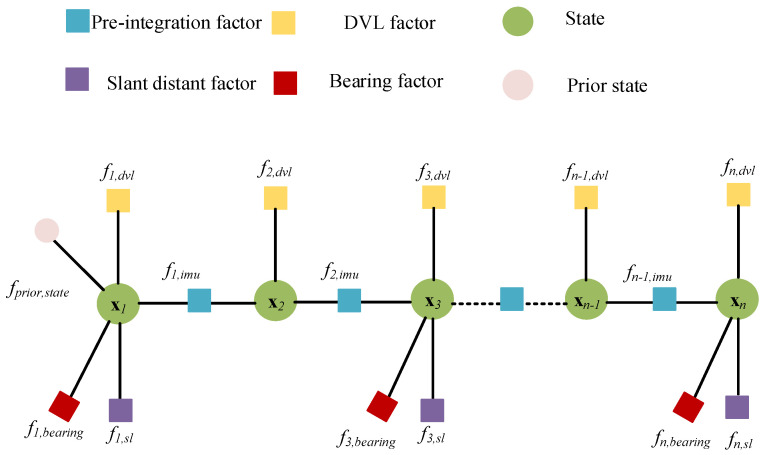
The graph model of the integrated navigation.

Based on the above FGO theory, we organized the graph model of the integrated navigation as Figure 2 follows:

The factors involved in the graph model will be analyzed in detail in the following. The algorithm is designed in two parts. One is the design of the measurement factors, including the IMU pre-integration factor, USBL factor, and DVL factor. With the design of the factors, the graph model of FGO can be conducted. The other is the design of the outlier elimination model. It can suppress the influence of outliers and improve the robustness of the system.

### 3.1. IMU Pre-Integration Factor

The output of IMU can be modeled as follows:

(11) $$\begin{array}{c} {\tilde{\omega }}_{ib}^{b}=\omega_{ib}^{b}+b_{g}+n_{g}\\ {\tilde{f}}^{b}=f^{b}+b_{a}+n_{a} \end{array}$$

where ${\tilde{\omega }}_{ib}^{b}$ and ${\tilde{\;f}}^{b}$ are the actual output of IMU. $b_{g}$ is the gyroscope bias, and $b_{a}$ is the accelerometer bias. $\omega_{ib}^{b}$ and $f^{b}$ are the ideal outputs of IMU. $n_{g}$ is the white noise of the gyroscope, and $n_{a}$ is the white noise of the accelerometer.

Assume that the interval of pre-integration is $\left[t_{k},t_{k+1}\right]$. The pre-integration measurements using the output of IMU can be expressed as follows:

(12) $$\{\begin{array}{c} \alpha_{bk+1}^{bk}=\iint_{t_{k}}^{t_{k+1}}C_{bt}^{bk}\left({\tilde{f}}^{b}-b_{a}\right)dt\\ \beta_{bk+1}^{bk}=\int_{t_{m}}^{t_{m+1}}C_{bt}^{bm}\left({\tilde{f}}^{b}-b_{a}\right)dt\\ \gamma_{bk+1}^{bk}=\int_{t_{k}}^{t_{k+1}}\frac{1}{2}\Omega \left({\tilde{\omega }}_{ib}^{b}-b_{g}\right)\gamma_{b_{t}}^{b_{k}}dt \end{array}$$

where:

(13) $$\Omega \left(\omega \right)=\left[\begin{array}{cc} -{\left[\omega \right]}_{\times } & \omega \\ -\omega^{T} & 0 \end{array}\right]$$

where ${\left[\omega \right]}_{\times }$ denotes the skew-symmetric matrix.

The position’s pre-integration is expressed as:

(14) $${\tilde{\alpha }}_{bk+1}^{bk}=C_{nk+1}^{bk}\left(P_{k+1}^{n}-P_{k}^{n}-V_{k}^{n}\Delta t_{k,k+1}-\frac{1}{2}g^{n}\Delta t_{k,k+1}^{2}\;+\frac{1}{2}\left(2\omega_{ie}^{n}+\omega_{en}^{n}\right)\times V_{k}^{n}\Delta t_{k,k+1}^{2}\right)$$

where $P_{k}^{n}$ denotes the position at epoch m. $V_{k}^{n}$ denotes the velocity at epoch m. $g^{n}$ denotes the gravity vector in the n-frame. $\Delta t_{k,k+1}$ is the time interval from $t_{k}$ to $t_{k+1}$. $\omega_{en}^{n}$ and $\omega_{ie}^{n}$ are expressed as follows:

(15) $$\begin{array}{c} \omega_{ie}^{n}={\left[\begin{array}{ccc} 0 & \omega_{ie}\cos L & \omega_{ie}\sin L \end{array}\right]}^{T}\\ \omega_{en}^{n}={\left[\begin{array}{ccc} -\frac{V_{N}}{R_{M}+h} & \frac{V_{E}}{R_{N}+h} & V_{E}\tan L/\left(R_{N}+h\right) \end{array}\right]}^{T} \end{array}$$

where $\omega_{ie}$ is the earth rotation rate.

The velocity pre-integration with respect to the states is expressed as:

(16) $${\tilde{\beta }}_{bk+1}^{bk}=C_{nk+1}^{bk}\left(V_{k+1}^{n}-V_{k}^{n}-g^{n}\Delta t_{k,k+1}\;\;\;\;\;\;\;\;\;\;\;\;\;+\left(2\omega_{ie}^{n}+\omega_{en}^{n}\right)\times \left(V_{k+1}^{n}+V_{k}^{n}\right)\frac{1}{2}\Delta t_{k,k+1}\right)$$

The attitude’s pre-integration with respect to the states is expressed as:

(17) $${\tilde{\gamma }}_{bk+1}^{bk}={\left({\left(q_{bk+1}^{nk+1}\right)}^{-1}⊗q_{nk}^{nk+1}⊗q_{bk}^{nk}\right)}^{-1}$$

where $q_{nm}^{nm+1}$ is updated as follows:

(18) $${\dot{C}}_{n}^{in0}=C_{n}^{in0}\left(\omega_{in}^{n}\times \right)$$

If the estimation of bias changes, the pre-integration measurements can be expressed as follows:

(19) $$\{\begin{array}{c} {\tilde{\alpha }}_{bk+1}^{bk}\approx {\tilde{\alpha }}_{bk+1}^{bk}+J_{bg}^{\alpha }\delta b_{g}+J_{ba}^{\alpha }\delta b_{a}\\ {\tilde{\beta }}_{bk+1}^{bk}\approx {\tilde{\beta }}_{bk+1}^{bk}+J_{bg}^{\beta }\delta b_{g}+J_{ba}^{\beta }\delta b_{a}\\ {\tilde{\gamma }}_{bk+1}^{bk}\approx {\tilde{\gamma }}_{bk+1}^{bk}⊗\left[\begin{array}{c} 1\\ \frac{1}{2}J_{bg}^{\gamma }\delta b_{g} \end{array}\right] \end{array}$$

where $J_{bg}^{\alpha }$,$\;J_{ba}^{\alpha }$,$\;J_{bg}^{\beta }$,$\;J_{ba}^{\beta }$,$\;J_{bg}^{\gamma }$ are the sub-matrix, which can be seen in [18].

Thus, the corresponding IMU pre-integration residuals can be expressed as:

(20) $$r_{imu}\left(Z_{imu,k},X\right)=\left[\begin{array}{c} \alpha_{bk+1}^{bk}-{\tilde{\alpha }}_{bk+1}^{bk}\\ \beta_{bk+1}^{bk}-{\tilde{\beta }}_{bk+1}^{bk}\\ 2{\left[{\left(q_{bk+1}^{nk+1}\right)}^{-1}⊗q_{nk}^{nk+1}⊗q_{bk}^{nk}⊗{\tilde{\gamma }}_{bk+1}^{bk}\right]}_{xyz}\\ b_{gk+1}-b_{gk}\\ b_{ak+1}-b_{ak} \end{array}\right]$$

### 3.2. USBL Factor

The USBL measurements contain the slant distance and bearing angles.

According to (2), the slant distance can be calculated as:

(21) $$\tilde{r}=\|C_{g}^{n}\left(t^{g}-p^{g}\right)\|$$

The bearing angles can be calculated as:

(22) $$\{\begin{array}{c} \tilde{\alpha }=acos\left(t_{u,x}/r\right)\\ \tilde{\beta }=acos\left(t_{u,y}/r\right) \end{array}$$

Thus, the USBL residuals can be expressed as:

(23) $$r_{usbl}\left(Z_{usbl,k},X\right)=\left[\begin{array}{c} \tilde{r}-r\\ \tilde{\alpha }-\alpha \\ \tilde{\beta }-\beta \end{array}\right]$$

### 3.3. DVL Factor

The DVL measures the speed in the b-frame. It has the following relationship with the AUV velocity:

(24) $$V^{n}=C_{b}^{n}V^{b}+n_{dvl}$$

where $n_{dvl}$ is the measurement noise, which obeys the Gaussian distribution.

Thus, the residuals of the DVL factor can be expressed as:

(25) $$r_{dvl}\left(Z_{dvl,k},X\right)=C_{n}^{b}V^{n}-V^{b}$$

Based on (20), (23) and (25), the optimization problem can be expressed as:

(26) $$\underset{x}{\min}\left\{\|r_{p}-H_{p}\|X_{\Sigma_{p}}^{2}+\underset{k\in \left[0,n\right]}{\sum }\|r_{imu}{\left(Z_{imu,k},X)\right\|}_{\Sigma_{imu,k}}^{2}\;\;\;\;\;\;+\underset{k\left[0,n\right]}{\sum }\|r_{usbl}{\left(Z_{usbl,k},X)\right\|}_{\Sigma_{usbl,k}}^{2}+\underset{k\in \left[0,n\right]}{\sum }\|r_{dvl}{\left(Z_{dvl,k},X)\right\|}_{\Sigma_{dvl,k}}^{2}\right\}$$

where $\Sigma_{imu,k}$, $\Sigma_{usbl,k}$, and $\Sigma_{dvl,k}$ are the measurement covariance. $\|r_{p}-H_{p}{X\|}_{\Sigma_{p}}^{2}$ is the prior factor, and $\Sigma_{p}$ is the prior covariance.

### 3.4. Outlier Elimination Algorithm

To reduce the influence of outliers, the residual processing method based on the maximum correlation entropy criterion is used in FGO.

The correlation entropy between two random variables **X** and **Y** can be defined as:

(27) V(X,Y)=E[κ(X,Y)]=∬κ(X,Y)F(X,Y)dxdy

where E[·] is the expectation. κ(X,Y) denotes the kernel function. F(X,Y) denotes the joint probability density function of the random variables X and Y.

The Gaussian function is selected as the Kernel function, which is as follows:

(28) $$\kappa \left(x_{i},y_{i}\right)=G_{\sigma }\left(e_{i}\right)=exp\left(\frac{e_{i}^{2}}{2\sigma^{2}}\right)$$

where ${\left\{x_{i},y_{i}\right\}}_{i=1}^{N}$ means the *N* samples satisfying the joint probability density F(X,Y). $e_{i}=x_{i}-y_{i}$. σ denotes the bandwidth of the kernel function.

The estimated value of correlation entropy can be expressed as:

(29) $$V\left(X,Y\right)=\frac{1}{N}\overset{N}{\underset{i=1}{\sum }}\kappa \left(x_{i},y_{i}\right)$$

In (26), $r_{usbl}\left(Z_{usbl,k},X\right)$ and $r_{dvl}\left(Z_{dvl,k},X\right)$ can be formulated as:

(30) $$\{\begin{array}{c} r_{usbl}\left(Z_{usbl,k},X\right)=Z_{usbl,k}-H_{usbk,k}X\\ r_{dvl}\left(Z_{dvl,k},X\right)=Z_{dvl,k}-H_{dvl,k}X \end{array}$$

where $Z_{usbl,k}$ and $Z_{dvl,k}$ are measurements. $H_{usbk,k}$ and $H_{dvl,k}$ are the Jacobian matrix.

The optimization function can be expressed as:

(31) $$\underset{x}{\min}\left\{\|r_{p}-H_{p}{X\|}_{\Sigma_{p}}^{2}+\underset{k\in \left[0,n\right]}{\sum }\|r_{imu}{\left(Z_{imu,k},X\right)\|}_{\Sigma_{imu,k}}^{2}\;+\underset{k\left[0,n\right]}{\sum }\|Z_{usbl,k}-H_{usbk,k}{X\|}_{\Sigma_{usbl,k}}^{2}\;+\underset{k\in \left[0,n\right]}{\sum }\|Z_{dvl,k}-H_{dvl,k}{X\|}_{\Sigma_{dvl,k}}^{2}\right\}$$

Let $e_{usbl,k}=\Sigma_{usbl,k}^{-1/2}\left(Z_{usbl,k}-H_{usbk,k}X\right)$,$\;e_{dvl,k}=\Sigma_{dvl,k}^{-1/2}\left(Z_{dvl,k}-H_{dvl,k}X\right)$. Then, (31) can be reformulated as follows:

(32) $$\underset{x}{\min}\left\{\|r_{p}-H_{p}{X\|}_{\Sigma_{p}}^{2}+\underset{k\in \left[0,n\right]}{\sum }\|r_{imu}{\left(Z_{imu,k},X\right)\|}_{\Sigma_{imu,k}}^{2}+\underset{k\left[0,n\right]}{\sum }\overset{l}{\underset{i=1}{\sum }}e_{usbl,ki}^{2}\;+\underset{k\in \left[0,n\right]}{\sum }\overset{m}{\underset{i=1}{\sum }}e_{dvl,ki}^{2}\right\}$$

where *l* and *m* are the dimensions of the measurement.

The Gaussian Kernel of the maximum entropy criterion is introduced into the measured part of the cost function.

(33) $$\underset{x}{\min}\left\{\|r_{p}-H_{p}{X\|}_{\Sigma_{p}}^{2}+\underset{k\in \left[0,n\right]}{\sum }\|r_{imu}{\left(Z_{imu,k},X)\right\|}_{\Sigma_{imu,k}}^{2}+\underset{k\left[0,n\right]}{\sum }\sigma^{2}\overset{l}{\underset{i=1}{\sum }}G_{\sigma }\left(e_{usbl,ki}\right)\;+\underset{k\in \left[0,n\right]}{\sum }\sigma^{2}\overset{m}{\underset{i=1}{\sum }}G_{\sigma }\left(e_{dvl,ki}\right)\right\}$$

Take the *USBL* and *DVL* measurement parts at epoch *k* as an example, and the derivative at X is:

(34) $$\overset{l}{\underset{i=1}{\sum }}G_{\sigma }\left(e_{usbl,ki}\right)e_{usbl,ki}\frac{\partial e_{usbl,ki}}{\partial X}+\overset{l}{\underset{i=1}{\sum }}G_{\sigma }\left(e_{dvl,ki}\right)e_{dvl,ki}\frac{\partial e_{dvl,ki}}{\partial X}=0$$

(34) can be reformulated as follows:

(35) $$H_{usbl,k}^{T}\Sigma_{usbl,k}^{-T/2}\psi_{usbl,k}e_{usbl,k}+H_{dvl,k}^{T}\Sigma_{dvl,k}^{-T/2}\psi_{dvl,k}e_{dvl,k}=0$$

where $\psi_{usbl,k}=diag\left[G_{\sigma }\left(e_{usbl,ki}\right)\right]$. $\psi_{dvl,k}=diag\left[G_{\sigma }\left(e_{dvl,ki}\right)\right]$.

Based on (35), (31) can be reformulated as follows:

(36) $$\underset{x}{\min}\left\{\|r_{p}-H_{p}{X\|}_{\Sigma_{p}}^{2}+\underset{k\in \left[0,n\right]}{\sum }\|r_{imu}{\left(Z_{imu,k},X)\right\|}_{\Sigma_{imu,k}}^{2}\;\;\;\;\;+\underset{k\left[0,n\right]}{\sum }\|Z_{usbl,k}-H_{usbk,k}{X\|}_{{\tilde{\Sigma }}_{usbl,k}}^{2}\;\;\;\;\;+\underset{k\in \left[0,n\right]}{\sum }\|Z_{dvl,k}-H_{dvl,k}{X\|}_{{\tilde{\Sigma }}_{dvl,k}}^{2}\right\}$$

where ${\tilde{\Sigma }}_{usbl,k}\;$= $\Sigma_{usbl,k}^{-T/2}\psi_{usbl,k}\Sigma_{usbl,k}^{-1/2}$,${\tilde{\Sigma }}_{dvl,k}\;$=$\;\Sigma_{dvl,k}^{-T/2}\psi_{dvl,k}\Sigma_{dvl,k}^{-1/2}$.

The influence of outliers on the estimation results can be eliminated by adjusting the covariance matrix ${\tilde{\Sigma }}_{usbl,k}$ and ${\tilde{\Sigma }}_{dvl,k}$.

In (36), the residual function $r_{imu}\left(Z_{imu,k},X\right)$ is from the *IMU* factor, as (20) shows. The residual function $Z_{usbl,k}-H_{usbk,k}X$ is from the *USBL* factor, as (23) shows, and $Z_{dvl,k}-H_{dvl,k}X$ is from the *DVL* factor, as (25) shows.

The integrated navigation algorithm is implemented by (36). It is a typical MAP problem. When the measurements of *DVL*, *USBL*, and *IMU* are obtained, the residual function can be formulated as (36). The object is to obtain the optimal solution of X by solving Equation (36). The state X contains the position, velocity, and attitude information of the vehicles.

The Ceres Solver [26] is used to solve the MAP problem. The Jacobian matrix of *USBL* and *DVL* are computed by the automatic derivative function of Ceres.

## 4. Simulation and Field Test

Simulation and field tests will be conducted in this section. First, a simulation test with different measurement noise values is conducted. The simulation test is necessary as the sensor data is ideal, and it can verify the accuracy of the model and algorithm. Second, a field test is conducted. The field test was carried out in the Yangtze River, and it can verify the effectiveness of the algorithm in the real world and prove its practicality. Thus, both tests are necessary.

All algorithms run on a laptop equipped with AMD Ryzen 7 5800U CPU, whose Radeon Graphics is 1.90 GHz and 16 G RAM.

### 4.1. Simulation Test

In this section, a series of simulation experiments are designed to verify the effectiveness and improvement of the proposed method.

Symbols used in the simulation experiment are listed as follows:

‘KF’ represents the traditional Kalman filter.

‘HKF’ denotes the robust Kalman filter based a Huber M-estimation.

‘FGO’ denotes the traditional FGO algorithm as described in [27].

‘RFGO’ denotes the proposed robust FGO algorithm based on the maximum correlation entropy criterion.

In the simulation, the bias of the gyroscope is 0.01°/h, and its random walk noise is $0.005{}^{\circ}/\sqrt{h}$. The bias of the accelerometer is 100 μg, and its random walk noise is $50\mathrm{ug}/\sqrt{\mathrm{Hz}}$. The bearing error of USBL is $0.2^{{}^{\circ}}$, and the slant distance error is 1.5 m. Assume that the DVL’s bottom track is available throughout the test and that 0.05 m/s random noise is added to the measurement. The output frequency of IMU, USBL, and DVL is 200 Hz, 1/2 Hz, and 1 Hz.

The measurement noise of the system is set as follows:

(37) $$\begin{array}{c} V_{2}\sim \{\begin{array}{cc} N\left(0,\;R_{v}\right) & w.p.\;\;0.95\\ N\left(0,\;100R_{v}\right) & w.p.\;\;0.05 \end{array}\\ V_{2}\sim \{\begin{array}{cc} N\left(0,\;R_{r}\right) & w.p.\;\;0.95\\ N\left(0,\;200R_{r}\right) & w.p.\;\;0.05 \end{array}\\ V_{1}\sim \{\begin{array}{cc} N\left(0,\;R_{a}\right) & w.p.\;\;0.95\\ N\left(0,\;20R_{a}\right) & w.p.\;\;0.05 \end{array} \end{array}$$

where $V_{1}$, $V_{2},$ and $V_{3}$ are the bearing, slant distance noise, and DVL velocity noise, respectively, which obey the Gaussian distribution. w.p. 0.95 denotes the normal data, which appears with probabilities of 95%, and w.p. 0.05 denotes the outlier‘s probability, which is 5% [28].

In the integrated navigation system, the initial attitude error is set as ${\left[\begin{array}{ccc} 0.05^{{}^{\circ}} & 0.05^{{}^{\circ}} & 0.1^{{}^{\circ}} \end{array}\right]}^{T}$. The initial velocity and position error are set as ${\left[\begin{array}{ccc} 0.1 & 0.1 & 0.1 \end{array}\right]}^{T}m/s$ and ${\left[\begin{array}{ccc} 1 & 1 & 3 \end{array}\right]}^{T}m$. The initial absolute position is set as ${\left[\begin{array}{ccc} 118.7718^{{}^{\circ}}E & 32.0575^{{}^{\circ}}N & 300m \end{array}\right]}^{T}$.

The measurement covariance matrixes of USBL and DVL are set to be the same in these methods, as shown below:

$R_{usbl}=diag(\left[\begin{array}{ccc} 2.25 & 1.2e-5 & 1.2e-5 \end{array}\right]$) and $R_{dvl}=0.01I_{3\times 3}$.

**Figure 3:**
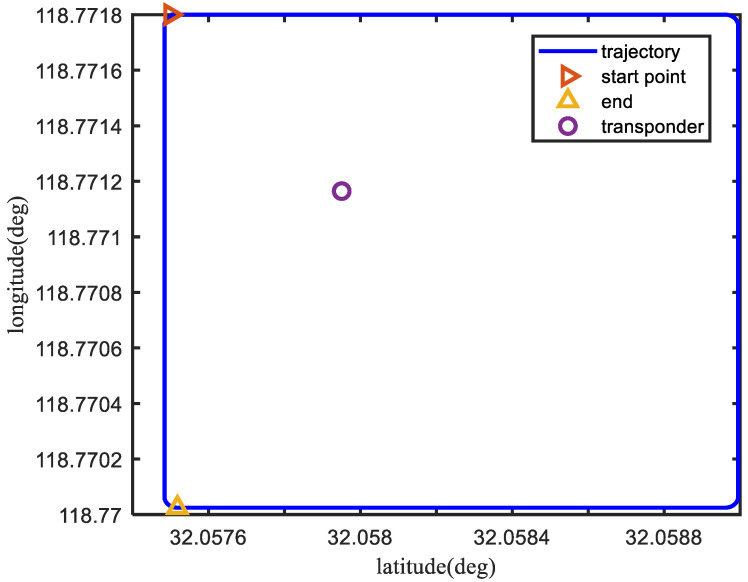
The AUV simulation trajectory.

The simulation trajectory of the vehicle is shown in Figure 3.

The velocity of the AUV is set as 2 m/s, and the transponder is shown as the purple circle in Figure 3.

**Figure 4:**
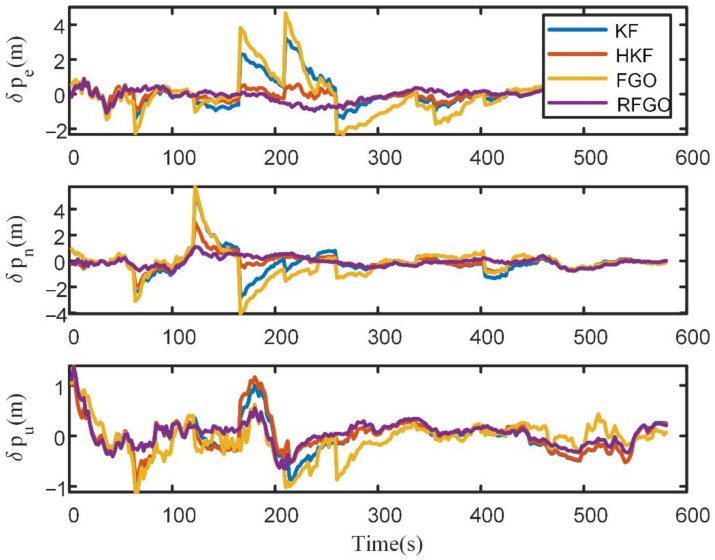
Position error of different algorithms.

The position errors of different algorithms are shown in Figure 4.

In Figure 4, the KF method without a robust algorithm suffers from the influence of outliers. The HKF method can suppress the influence of outliers. The Huber-based approach is limited in its ability to handle large outliers. Thus, it shows a worse performance than the proposed method. Even the traditional FGO method shows a worse performance than the KF method, as the yellow line shows. This is because the INS mechanization in [27] is simplified, with the angular rate $\omega_{en}^{n}$ being ignored. The estimation accuracy is not as high as that of the Kalman filtering method in high-precision inertial navigation. The proposed RFGO method shows a superior performance to the Kalman-filter-based method due to multiple iterations and a joint optimization of historical data.

In order to quantitatively analyze the estimation results, RMSE is used as the performance metric.

The RMSE results of different methods are listed in Table 1.

In Table 1, RFGO performs best among these methods. The position accuracy is improved by 56.9%, 11.8%, and 70.4% compared with the KF, HKF, and FGO in the east direction, respectively. It is also improved by 62.4%, 35.4%, and 67.8% in the north direction and by 23.4%, 22.2%, and 28.8% in the up direction.

To make the results more convincing, we conducted statistics on the results of 50 independent experiments.

The RMSE of the Monte Carlo simulation test is used as the performance metric.

**Figure 5:**
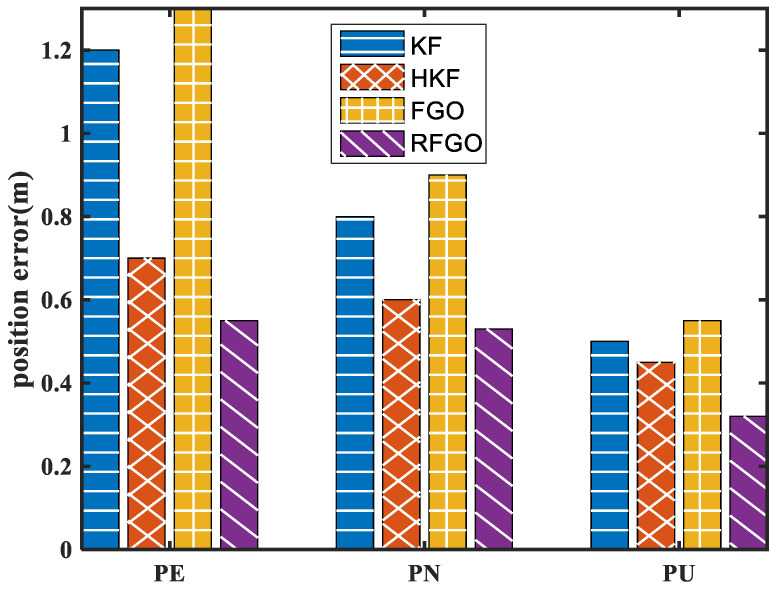
Position error of different algorithms in Monte Carlo simulation.

The error bar of different algorithms is shown in Figure 5.

In Figure 5, the proposed method has the highest accuracy and the most obvious performance improvement. In the case of nonlinearity and outliers, the advantage of our method is more obvious.

### 4.2. Field Test

In this section, a field test is designed to verify the effectiveness and improvement of the proposed method. The field test was carried out in the Yangtze River.

**Figure 6:**
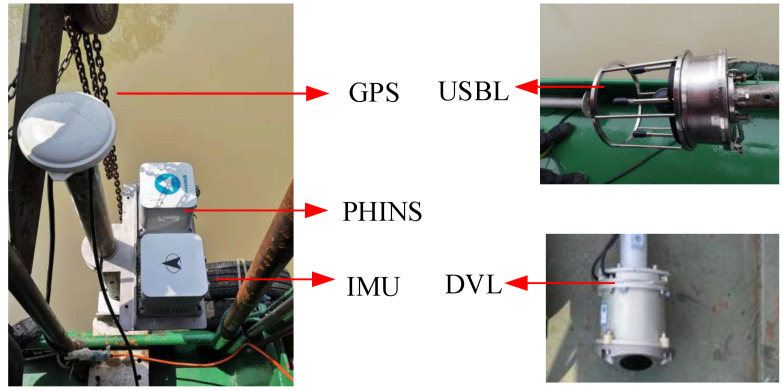
Diagram of the field test equipment.

The equipment used in the test includes the USBL, DVL, IMU, and RTK GPS/PHINS integrated system, where the output of the RTK GPS/PHINS integrated system is used as the true value. As Figure 6 shows:

In the field test, the transponder position and the installation error of USBL [29], as well as the scale factor of DVL [30], are calibrated in advance.

The performance of the sensors is as Table 2 follows.

In the field test, the initial navigation errors are set as follows:

Initial position error: $\left[\begin{array}{ccc} 0.1 & 0.1 & 0.1 \end{array}\right]m$; Initial velocity error: $\left[\begin{array}{ccc} 0.1 & 0.1 & 0.1 \end{array}\right]m/s$; Initial attitude error: $\left[\begin{array}{ccc} 0.05 & 0.05 & 0.1 \end{array}\right]deg$;

The measurement covariance matrixes of USBL and DVL are set to be the same in these methods, as shown below:

$R_{usbl}=diag(\left[\begin{array}{ccc} 1 & 7e-4 & 7e-4 \end{array}\right]$) and $R_{dvl}=0.1I_{3\times 3}$.

**Figure 7:**
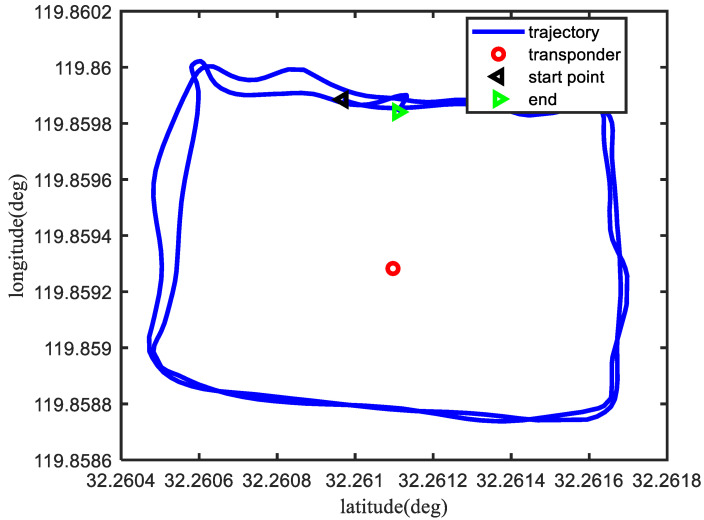
The vehicle trajectory in the field test.

The vehicle trajectory in the field test is shown in Figure 7.

**Figure 8:**
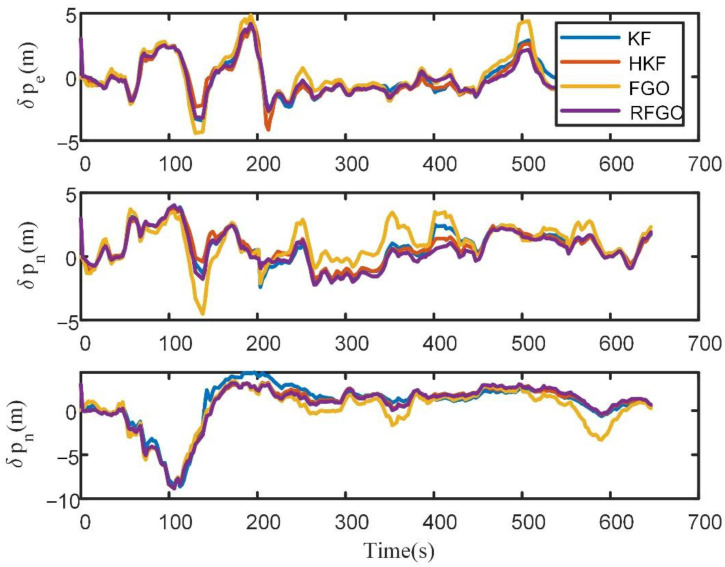
Position error of different algorithms in the field test.

The position error of different algorithms is shown in Figure 8.

In Figure 8, due to multiple iterations, the joint optimization of historical data, and the robust module based on the maximum correntropy criterion, the RFGO method outperforms the traditional Kalman-filter-based method. The traditional FGO method performs the worst due to the simplified INS mechanization and lack of a robust module. To quantitatively analyze the estimation results, RMSE is used as the performance metric.

The RMSE results of different methods are listed in Table 3.

The position errors of the RFGO method are significantly smaller than those of the Kalman filtering algorithm and the traditional FGO method. The proposed method has a higher positioning accuracy.

**Figure 9:**
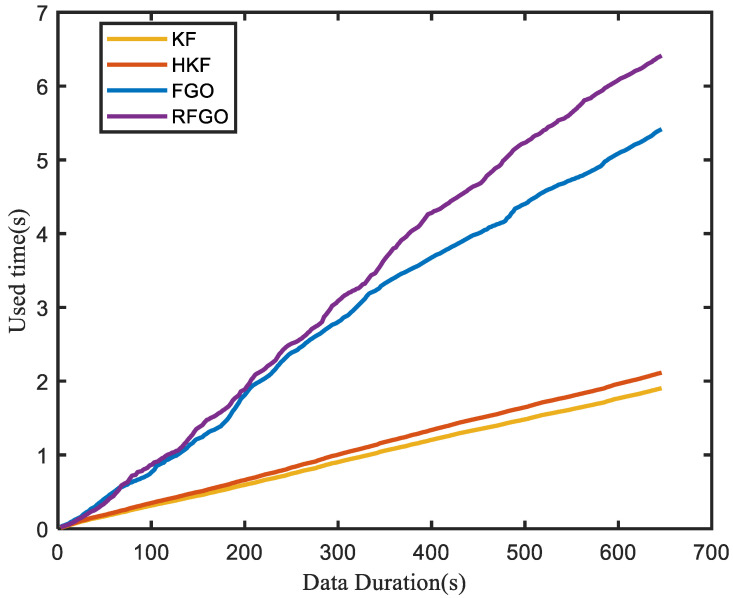
Execution time among different algorithms.

Computational burden is also one of the factors that reflect the performance of the algorithm. The execution time of different algorithms is an indicator of the computational burden, as paper [22] shows. To show the computational load of these methods, a comparison of the execution time among different algorithms is given in Figure 9.

It can be seen from Figure 9 that since only the current state is processed, the computational burden of the KF and HKF methods is minimal. The proposed FGO method is more complex than the FGO method, as the fault detection module is added. Although the proposed method is the most computationally intensive, it has the best localization performance and is an optional solution as long as the computer’s performance is adequate.

## 5. Conclusions

Underwater multi-sensor fusion is always the key to achieving a high-precision navigation and positioning of AUV. Different from the traditional filtering-based fusion scheme, we propose a factor graph optimization scheme, which is suitable for underwater INS/USBL/DVL integrated navigation systems. Multiple iterations and the re-linearization of FGO enable it to better solve the nonlinear problems of the system and improve the positioning accuracy. Considering the influence of outliers in the integrated navigation system, a robust fusion algorithm based on the maximum correlation entropy criterion in the FGO scheme is proposed. Numerical simulations and field tests demonstrate that the proposed scheme has a higher positioning accuracy and better robustness.

## Figures and Tables

**Table 1:** Comparison of the positioning error in the simulation.

	PE(m)	PN(m)	PU(m)
KF	0.79	0.96	0.34
HKF	0.34	0.56	0.34
FGO	1.14	1.12	0.36
RFGO	0.30	0.36	0.26

PE, PN, and PU denote the position error in east, north, and up directions.

**Table 2:** Parameters of the sensors.

Parameters			Value
IMU	Gyroscope	Constant	<0.01°/hr
		Random	$<0.005{}^{\circ}/\sqrt{\mathrm{hr}}$
	Accelerometer	Constant	$<5\times 10^{-4}\;g$
		Random	100 ug
USBL	Positioning error		0.1 m + 1%r
	Output frequency		1/2 Hz
DVL	Velocity tracking accuracy		±0.5%±5 mm/s
	Output frequency		1 Hz
PHINS/GPS	Positioning error		0.02–0.05 m

**Table 3:** Comparison of the positioning error in the test.

	PE(m)	PN(m)	PU(m)
KF	1.33	1.54	2.57
HKF	1.32	1.46	2.54
FGO	1.59	1.72	2.60
RFGO	1.26	1.40	2.44

## Data Availability

Not applicable.
